# Prognostic performance of cardiogenic shock 4 proteins prediction model in infarct-related cardiogenic shock

**DOI:** 10.1093/eschf/xvag010

**Published:** 2026-01-20

**Authors:** Danilo Obradovic, Lisa Schulz, Goran Loncar, Norman Mangner, Axel Linke, Uwe Zeymer, Steffen Desch, Janine Pöss, Anne Freund, Hans-Josef Feistritzer, Petra Büttner, Holger Thiele

**Affiliations:** Department for Internal Medicine and Cardiology, Heart Centre Dresden, Faculty of Medicine and University Hospital Carl Gustav Carus, TUD Dresden University of Technology, Fetscherstr. 76, Dresden 01307, Germany; Cardiology Department, Heart Center Leipzig at Leipzig University and Leipzig Heart Science, Leipzig, Germany; Institute for Cardiovascular Diseases ‘Dedinje’, University of Belgrade, Belgrade, Serbia; Faculty of Medicine, University of Belgrade, Belgrade, Serbia; Cardiology Department, Heart Center Leipzig at Leipzig University and Leipzig Heart Science, Leipzig, Germany; Cardiology Department, Heart Center Leipzig at Leipzig University and Leipzig Heart Science, Leipzig, Germany; Department of Cardiology and Angiology, Faculty of Medicine, University Heart Center Freiburg-Bad Krozingen, University of Freiburg, Feiburg, Germany; Institut für Herzinfarktforschung, Ludwigshafen, Germany; Cardiology Department, Heart Center Leipzig at Leipzig University and Leipzig Heart Science, Leipzig, Germany; Cardiology Department, Heart Center Leipzig at Leipzig University and Leipzig Heart Science, Leipzig, Germany; Cardiology Department, Heart Center Leipzig at Leipzig University and Leipzig Heart Science, Leipzig, Germany; Cardiology Department, Heart Center Leipzig at Leipzig University and Leipzig Heart Science, Leipzig, Germany; Cardiology Department, Heart Center Leipzig at Leipzig University and Leipzig Heart Science, Leipzig, Germany; Cardiology Department, Heart Center Leipzig at Leipzig University and Leipzig Heart Science, Leipzig, Germany

**Keywords:** Cardiogenic shock, Acute myocardial infarction, CS4P risk score, Mortality prognostication

## Abstract

**Introduction:**

The aim of this analysis was to evaluate the prognostic features of the cardiogenic shock 4 proteins (CS4P) biomarker-based risk score in patients with cardiogenic shock (CS), presenting with ST-segment elevation myocardial infarction (STEMI) vs non-ST-segment elevation myocardial infarction (NSTEMI), with and without cardiopulmonary resuscitation (CPR).

The CS4P risk score, validated in cohorts of CS patients with both acute coronary syndrome (ACS) and non-ACS aetiologies, showed advanced predictive metrics compared with other contemporary risk prediction scores for CS. However, there is lack of data concerning the prognostic performance of the CS4P score among CS patients with different forms of ACS.

**Methods:**

The present analysis is a post-hoc analysis of the randomized CULPRIT-SHOCK trial. The primary outcome was a composite of mortality or necessity for renal replacement therapy at 30-day follow-up. Cardiogenic shock 4 proteins markers were determined in serum using ELISA assays.

**Results:**

Of the 412 patients with CS included in this study, 240 (58.3%) patients had STEMI and 172 (41.7%) patients had NSTEMI. In CS patients presenting with STEMI, CS4P score exhibited better prognostication of the primary outcome compared with patients with NSTEMI [area under the curve (AUC) 0.74, 95% confidence interval (CI) 0.67–0.80 vs AUC 0.69, 95% CI 0.61–0.77; *P* = .05). Further, CS4P score displayed a higher prognostic performance in STEMI patients who had not undergone CPR prior to enrolment as compared with STEMI patients with preceding CPR (AUC 0.78; 95% CI 0.65–0.84 vs AUC 0.70, 95% CI 0.62–0.79; *P* < .001). Cardiogenic shock patients in the highest tertile of the CS4P risk score showed higher mortality rates within 30 days compared to those in the lowest tertile (hazard ratio 1.42, 95% CI 1.11–1.82; *P* = .005).

**Conclusion:**

The CS4P score provides acceptable short-term mortality risk stratification among patients with CS due to acute myocardial infarction. The CS4P prediction model exhibits superior prognostication among CS patients with STEMI as compared to NSTEMI and in STEMI patients without CPR prior to hospital presentation.

## Graphical Abstract

**Graphical Abstract:**
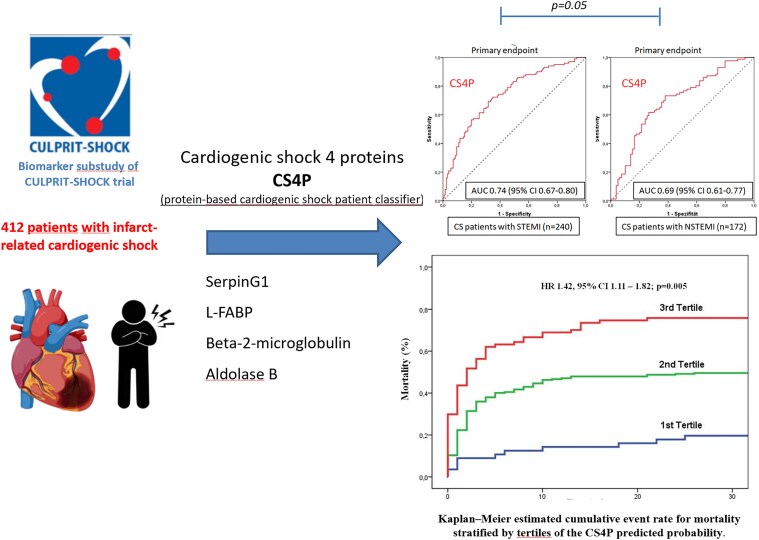

## Introduction

Cardiogenic shock (CS) remains one of the most severe complications of acute myocardial infarction (AMI) and is associated with high intrahospital mortality.^1^ Management of patients admitted with CS remains challenging despite progress in therapeutic strategies, including mechanical circulatory support.^2^

In order to optimize clinical management of CS patients, estimation of the individual patient's prognosis represents the cornerstone of initial diagnostic evaluation.^3^ A wide array of underlying pathologies in CS makes prognostication of its clinical outcomes challenging. Several risk scores have been developed to predict adverse outcome and mortality in CS patients. Although some of these scores are suitable to predict mortality risk in CS, their application depends on demanding and partly subjective clinical variables, such as angiographic and invasive haemodynamic data, which limits their wider clinical applicability.^4^

The utilization of circulatory biomolecules as surrogate markers of distinctive and disease specific pathophysiological processes is a useful tool in several cardiovascular conditions. Blood is accessible at any time and biomarkers, if standardized and established, can be easily and highly reproducibly measured at low costs. Thus, biomarker-based clinical algorithms offer significant benefits compared with more complex decision making tools, involving application of advanced and cost-intensive diagnostic and imaging procedures.

Bayés-Genís *et al*. developed the biomarker-based cardiogenic shock 4 proteins (CS4P) score based on liver-type fatty acid-binding protein (FABP1), beta-2-microglobulin (B2M), fructose-bisphosphate aldolase B (Aldolase B) and serpin G1 for short-term mortality in CS patients. The authors found that CS4P demonstrates 83% sensitivity and 75% specificity in prognostication of 90-day mortality in patients of non-pre-specified CS aetiology.^5^ However, there is a lack of evidence concerning the prognostic performance of the CS4P model among CS patients with different forms of AMI. Furthermore necessity for cardiopulmonary resuscitation (CPR) during initial CS stages significantly impairs outcome of patients, and it remains not fully determined whether CPR influences the prognostication performance of biomarker-based models in CS patients.

Based on these considerations, the aim of this analysis was to test the prognostication features of the CS4P model in CS patients with ST-segment elevation myocardial infarction (STEMI) vs non-ST-segment elevation myocardial infarction (NSTEMI) with further assessment of CPR influence.

## Methods

### Study population

The present analysis is a post-hoc sub-study of the randomized CULPRIT-SHOCK trial. Design and main results were published previously.^6^ In brief, the CULPRIT-SHOCK trial was a multicentre international randomized study including 706 patients with infarct-related CS and multivessel coronary artery disease. Immediate multivessel percutaneous coronary intervention (PCI) was compared with PCI of the culprit lesion only (with the option of staged revascularization at a later time point) with a composite primary endpoint of all-cause mortality or severe renal failure leading to renal replacement therapy within 30 days after randomization. Percutaneous coronary intervention of the culprit lesion only was superior to immediate multivessel PCI. The study was conducted according to the Declaration of Helsinki and written informed consent including blood sampling for laboratory analyses was obtained from study participants.

**Figure 1:**
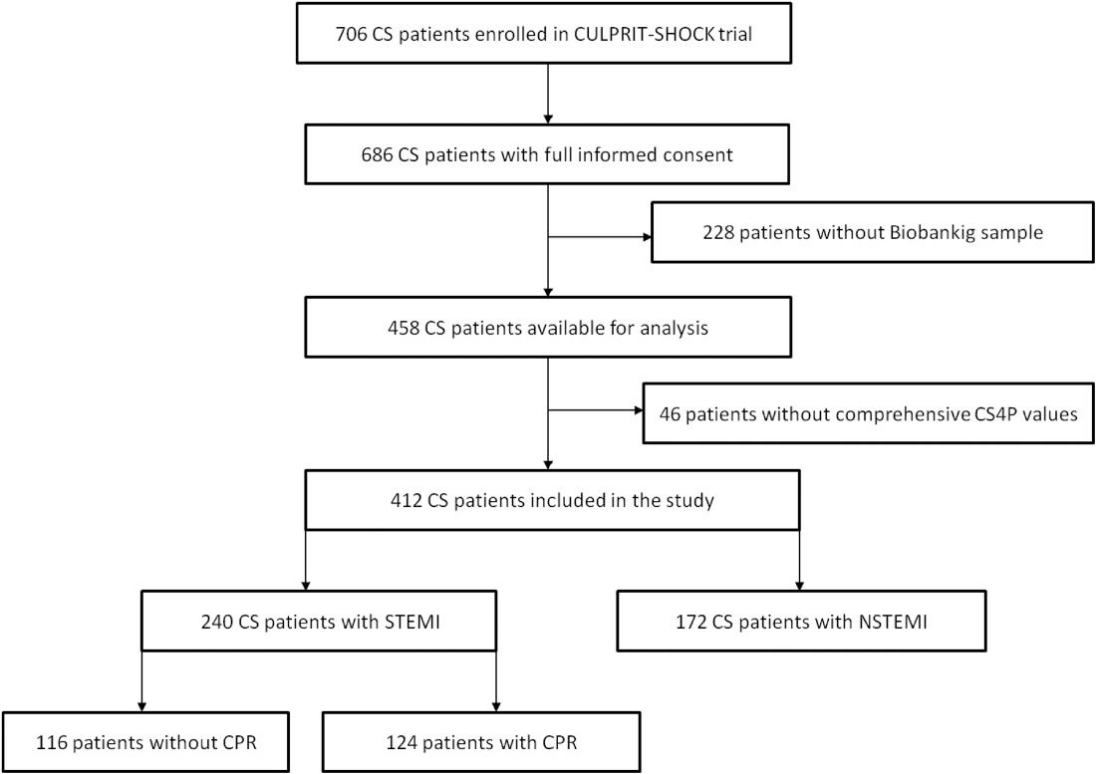
Study flow chart. CS, cardiogenic shock

Only patients with complete information concerning clinical status of study interest, as well as complete follow-up at 30 days were included in the present sub-analysis. Out of all patients included in the CULPRIT-SHOCK trial, blood samples from 412 patients were available for the current study (*Figure 1*). The blood was collected after enrolment of patients in the study prior to any respective study intervention.

### Cardiogenic shock 4 proteins biomarker profile

Cardiogenic shock 4 proteins markers were determined in serum. ELISA assays were used to detect Serpin G1 (order number: ELH-SERPING1, RayBiotech, Norcross, USA), FABP1 (order number: CSB-E13455h, Cusabio, Houston, USA), and Aldolase B (order number: SED319Hu, Cloud-Clone Corp., Katy, USA) and a LuminexXMAPassay was used to detect B2M (EPX010-12414-901, ThermoFisher, Waltham, USA). Pre-tests were done to determine sample dilutions and serum was diluted 1:2 for B2M and Aldolase B, 1:200 for L-FABP and 1:600 for Serpin G1. If sample diluent was provided by the assay manufacturer it was used, otherwise dilutions were prepared with phosphate buffered saline pH 7.2. All assays were processed as recommended by the manufacturer. Raw data were inspected and data out of range were excluded. To address inter-plate variations the individual sample data were normalized to the median of all samples on the specific plate.

### Definition of outcomes

The primary outcome was a composite of mortality or necessity for renal replacement therapy at 30-day follow-up. Secondary outcomes represented the individual components of the primary outcome. The study participants were classified as having STEMI or NSTEMI according to current guideline recommendations at the time of study conduction.^7^ Cardiopulmonary resuscitation was defined and conducted in accordance with the 2015 European Resuscitation Council guidelines. This definition aligns with contemporary resuscitation standards applicable during the study period.^8^

### Statistical analysis

Categorical variables are presented as number and percentages. Continuous data are presented as mean with standard deviation. The Kolmogorov–Smirnov test was used to test for normal distribution. For categorical variables, χ^2^ or Fisher exact test, as well as Mann–Whitney *U* and Kruskal–Wallis tests, depending on sample size, were applied. For continuous variables unpaired two-sample *t*-tests or one-way analysis of variance were calculated. Receiver operating curves (ROC) analysis was applied to evaluate the accuracy of explanatory variables for dichotomous outcome. Youden Index depicted optimal cut-off values from the ROC for prognostic purposes, and areas under the curves (AUC) were compared by the DeLong method. The relative contribution of every individual CS4P biomarker to outcome prediction was calculated by dividing logistic regression results through the standard deviation of the respective biomarker. Kaplan–Meier curves were used to visualize the mortality of the patients after they were stratified by tertiles of predicted mortality using the CS4P score. Hazard ratios (HR) with 95% confidence intervals (CI) were calculated using Cox proportional hazards regression. A two-sided *P*-value of .05 was considered statistically significant for all tests. Statistical analyses and description of baseline characteristics were done with SPSS Statistics® 27 (IBM, Armonk, NY, USA), GraphPad Prism software version 5.04 (GraphPad Software Inc., La Jolla, CA, USA), and R version 4.5.1 (R Core Team, 2024).

## Results

Patient demographic and procedural characteristics at baseline are shown in *Table 1*. Of the 412 CS patients included in this study, 240 (58.3%) patients had STEMI and 172 (41.7%) patients had NSTEMI (*Figure 1*). Cardiogenic shock patients presenting with NSTEMI had more often a history of a hypertension (69.1 vs 53.7%, *P* = .002), diabetes mellitus (41.3 vs 26.6%, *P* = .003), previous PCI (25.0 vs 14.1%, *P* = .007), previous coronary artery bypass grafting (11.0 vs 0.8%, *P* < .001), and atrial fibrillation (15.7 vs 6.6%, *P* = .005) compared with CS patients with STEMI (*Table 1*). There were no significant differences regarding mortality (45.0 vs 51.7%, *P* = .19), frequency of renal replacement therapy (13.7 vs 18.0%, *P* = .24) and primary endpoint (58.7 vs 69.7%; *P* = .34) at 30-day follow-up between CS patients with STEMI vs NSTEMI.

**Table 1:** Demographic and procedural characteristics of study cohort

Baseline characteristics of CS patients (*n* = 412)	STEMI (*n* = 240)	NSTEMI (*n* = 172)	*P*
Age, years (mean ± SD)	68 ± 11	70 ± 10	.79
Male—no./total no. (%)	177/240 (73.7%)	133/172 (77.3%)	.42
BMI, kg/m^2^ (mean ± SD)	27.4 ± 4.1	27.7 ± 4.4	.43
Hypertension—no./total no. (%)	129/240 (53.7%)	119/172 (69.1%)	.002
Diabetes mellitus—no./total no. (%)	64/240 (26.6%)	71/172 (41.3%)	.003
Previous myocardial infarction—no./total no. (%)	33/240 (13.7%)	28/172 (16.2%)	.48
Dyslipidaemia—no./total no. (%)	64/240 (26.6%)	62/172 (36.0%)	.05
Previous PCI—no./total no. (%)	34/240 (14.1%)	43/172 (25.0%)	.007
Known peripheral artery disease—no./total no. (%)	23/240 (9.5%)	21/172 (12.2%)	.42
Previous CABG—no./total no. (%)	2/240 (0.8%)	19/172 (11.0%)	<.001
Atrial fibrillation—no./total no. (%)	16/240 (6.6%)	27/172 (15.7%)	.005
Previous stroke—no./total no. (%)	11/240 (4.5%)	14/172 (8.1%)	.15
Known chronic kidney failure (GFR < 30 ml/min)—no./total no. (%)	13/240 (5.4%)	16/172 (9.3%)	.17
CPR 24 h before randomization—no./total no. (%)	124/240 (51.6%)	92/172 (53.4%)	.76
Serum lactate pre-PCI, mmol/l (mean ± SD)	4.8 ± 2.9	4.5 ± 2.7	.42
Creatinine, µmol/l (total no.)	120 ± 61 (227)	130 ± 55 (166)	.78
Acute left ventricular ejection fraction, % (mean ± SD)	34 ± 14	34 ± 13	.17
Mechanical circulatory support (any)—no./total no. (%)	64/240 (23.7%)	49/172 (28.4%)	.68
Mechanical ventilation—no./total no. (%)	189/240 (78.7%)	146/172 (84.8%)	.12
Catecholamine therapy—no./total no. (%)	211/240 (87.9%)	156/172 (90.6%)	.37
**Culprit lesion only PCI—no./total no. (%)**	**123/240 (51.2%)**	**90/172 (52.3%)**	.**68**
Mortality at 30 days—no./total no. (%)	108/240 (45.0%)	89/172 (51.7%)	.19
Renal replacement therapy at 30 days—no./total no. (%)	33/240 (13.7%)	31/172 (18.0%)	.24

Continuous variables are presented as mean ± standard deviation; categorical variables are presented as *n*/*n* (%).

BMI, body mass index; CABG, coronary artery bypass grafting; CS, patients with cardiogenic shock; CPR, cardiopulmonary resuscitation; NSTEMI, non-ST-elevation myocardial infarction; PCI, percutaneous coronary intervention; STEMI, ST-elevation myocardial infarction.

**Figure 2:**
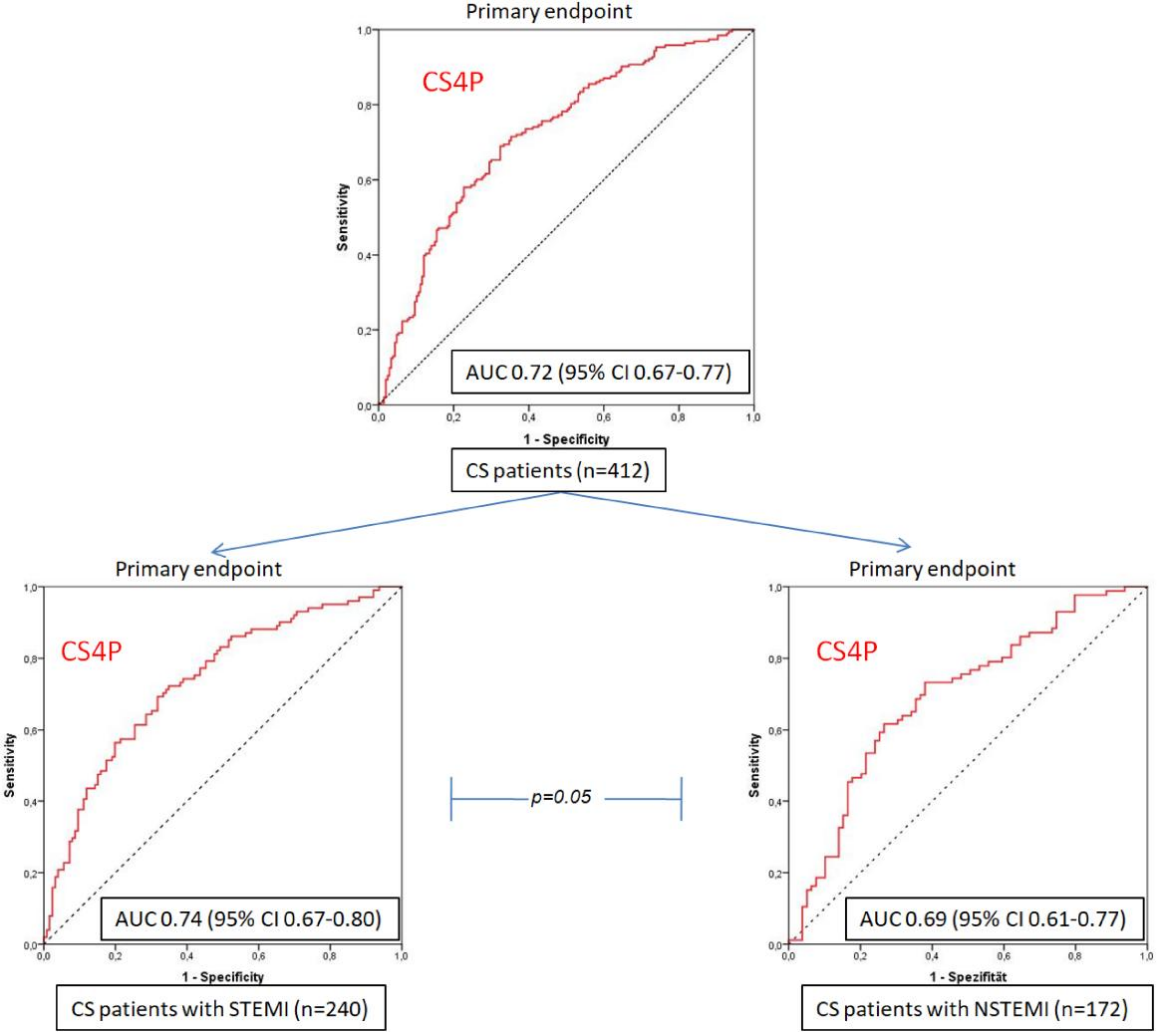
Receiver operating curves analysis of cardiogenic shock 4 proteins prognostication of primary study outcome in the entire study cohort and stratified for cardiogenic shock patients presenting with ST-segment elevation myocardial infarction vs non-ST-segment elevation myocardial infarction

**Figure 3:**
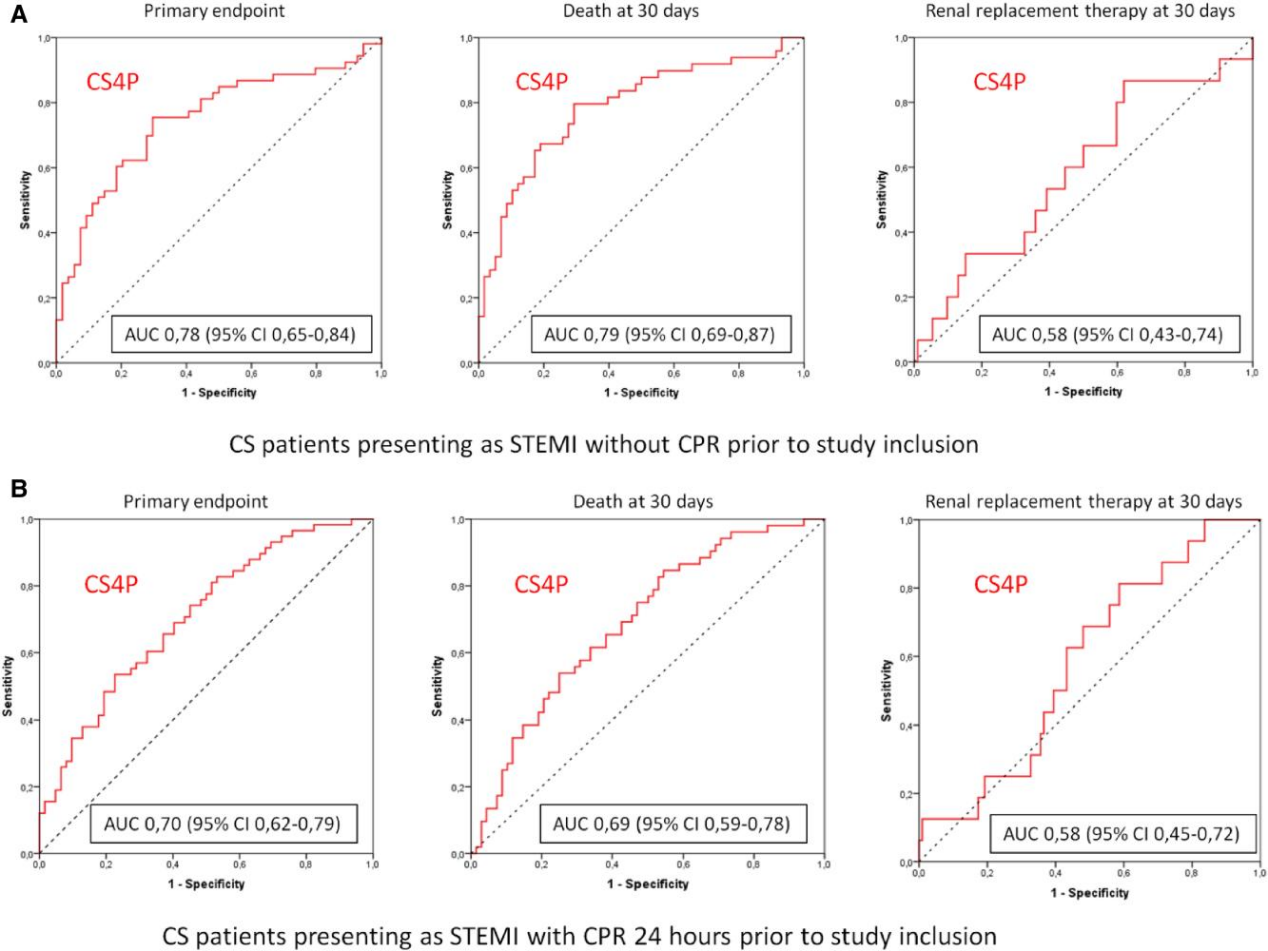
Receiver operating curves analysis of the cardiogenic shock 4 proteins prognostication of the primary outcome after 30 days for death or renal replacement therapy among cardiogenic shock patients presenting as ST-segment elevation myocardial infarction with and without cardiopulmonary resuscitation

**Figure 4:**
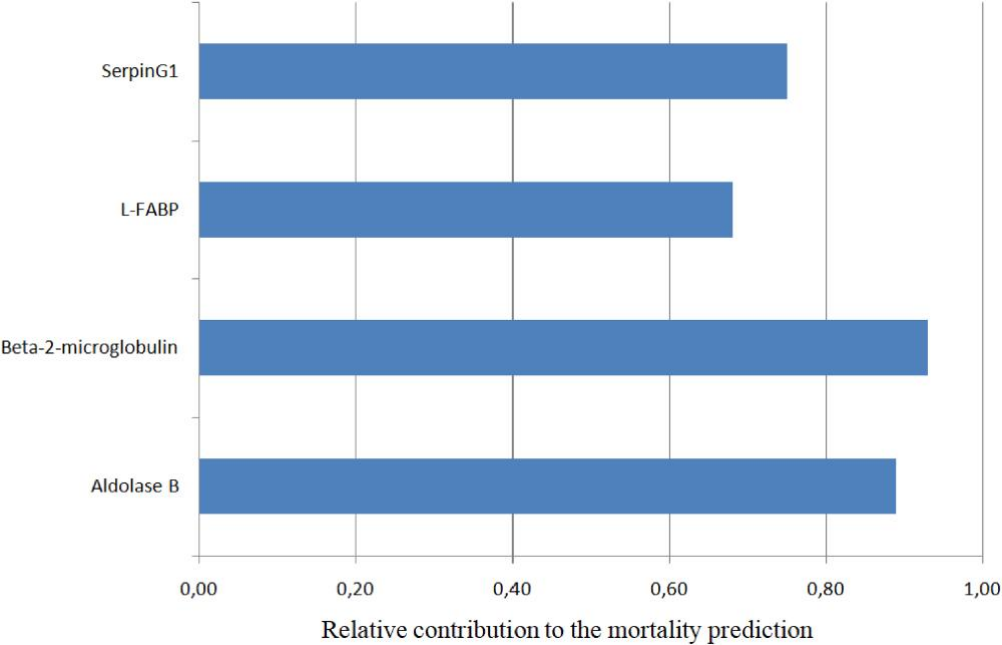
Relative contribution of the four biomarkers in the cardiogenic shock 4 proteins score to mortality prediction in cardiogenic shock patients

**Figure 5:**
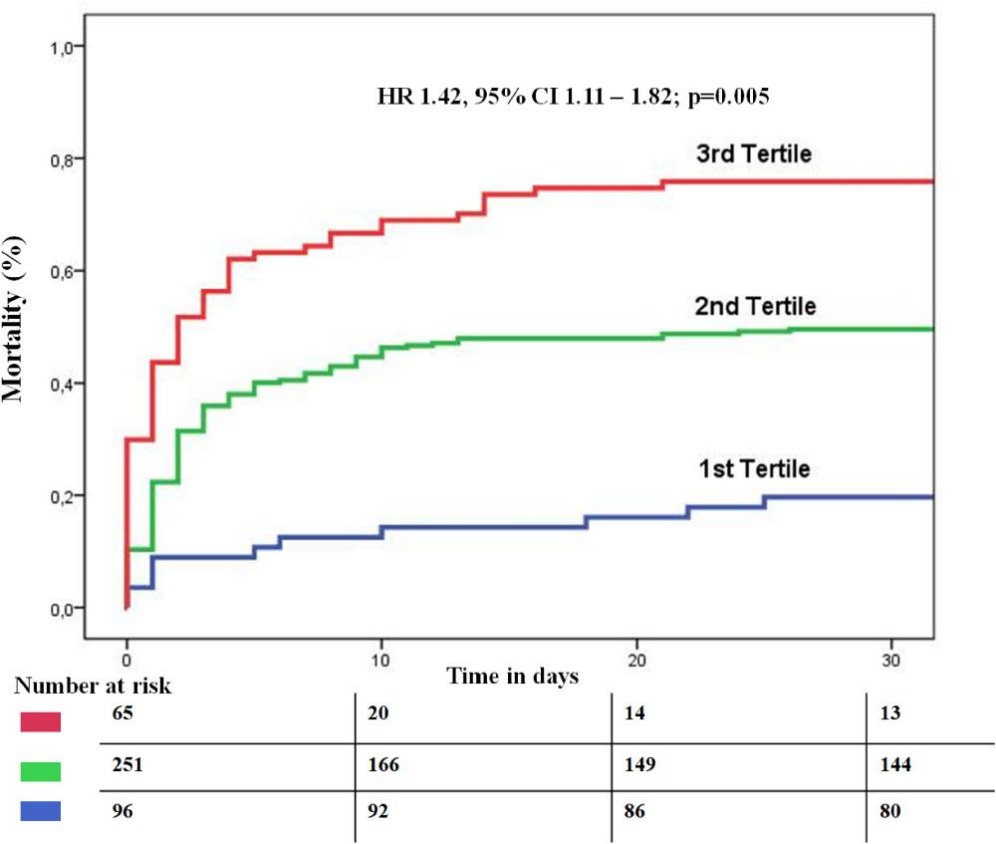
Kaplan–Meier estimated cumulative event rate for the 30 day mortality depicted for patients stratified by tertiles of the cardiogenic shock 4 proteins predicted probability

The CS4P score presented overall good prognostic performance (AUC 0.72, 95% CI 0.67–0.77; sensitivity 75% and specificity 69%) in the complete cohort. In CS patients presenting with STEMI, CS4P exhibited significantly better prognostication of the primary outcome compared with CS patients with NSTEMI (AUC 0.74, 95% CI 0.67–0.80 vs AUC 0.69, 95% CI 0.61–0.77; *P* = .05; *Figure 2*). Furthermore, in STEMI patients the CS4P score exhibited a significantly higher prognostic performance when patients had no CPR prior to study inclusion as compared with STEMI patients with preceding CPR (AUC 0.78; 95% CI 0.65–0.84 vs AUC 0.70, 95% CI 0.62–0.79; *P* < .001; *Figure 3*). In CS patients with STEMI CS4P generally exhibited significantly higher accuracy in prognostication of mortality (without CPR: 0.74, with CPR 0.63) than for renal replacement therapy at 30-day follow-up (with CPR: 0.52, without CPR: 0.53; *Table 2*). The prognostic performance of the CS4P score in NSTEMI CS patients is presented in the Supplementary material. The relative contribution of each CS4P biomarker component to predicting the primary endpoint in the entire study cohort ranged from 0.68 to 0.93, with B2M showing the highest value (*Figure 4*). Cardiogenic shock patients in the highest tertile of the by CS4P predicted risk, had significantly higher mortality rates within 30 days compared with CS patients in the lowest tertile (HR 1.42, 95% CI 1.11–1.82; *P* = .005) (*Figure 5*).

**Table 2:** Prognostic performance of CS4P in STEMI patients with and without CPR prior to study inclusion

Prognostic performance of CS4P	Sensitivity	Specificity	PPV	NPV	Accuracy	*P*
**CS patients presenting with STEMI without CPR prior to study inclusion**						
Composite endpoint	76%	70%	0.71	0.74	0.72	<.001
Death at 30 days	80%	71%	0.69	0.80	0.74	<.001
Renal replacement therapy at 30 days	68%	50%	0.20	0.90	0.52	.26
**CS patients presenting with STEMI undergoing CPR within 24 h prior to study inclusion**						
Composite endpoint	62%	63%	0.61	0.64	0.63	.006
Death at 30 days	63%	62%	0.56	0,69	0.63	.006
Renal replacement therapy at 30 days	62%	53%	0.17	0.90	0.54	.25

CS, patients with cardiogenic shock; CPR, cardiopulmonary resuscitation; NSTEMI, non-ST-elevation myocardial infarction; NPV, negative predictive value; PPV, positive predictive value; STEMI, ST-elevation myocardial infarction.

## Discussion

In this study we observed: (i) The CS4P score enables clinically relevant prognostication of death at 30-day follow-up in patients with CS due to AMI; (ii) The CS4P score performs significantly better in CS patients presenting with STEMI vs NSTEMI; (iii) The CS4P prediction model provided better prediction accuracy in CS patients presenting with STEMI who did not undergo CPR within 24 h prior to study inclusion compared with resuscitated STEMI patients; (iv) The CS4P score showed better prognostication in terms of 30-day mortality in comparison to necessity for renal replacement therapy.

Several scores predicting clinical outcome in severely ill patients in the intensive care units have been developed in previous decades (such as APACHE, SAPS II, and SAPS 3). However, the first specific prognostic systems for CS exhibited obvious shortcomings due to small sample sizes of validation cohorts and development predominantly in the pre-PCI era.^9–11^

More recently, the IABP-SHOCK II (intra-aortic balloon pump in cardiogenic shock) score was one of the first multivariable prediction score for patients with CS due to AMI that was internally and externally validated in two large CS patient populations.^12^ The CardShock score for mortality prediction was also developed in a large CS cohort; however, the score was not specifically related to AMI as aetiology of CS.^13^ Both prediction models represented pioneering work and were for the first time suitable for reliable and validated risk stratification in CS patients.^4^ However, due to the emergent nature of CS management and altered mental status of haemodynamically compromised patients with CS, some of the clinical variables important for IABP-SHOCK II and CardShock prognostication (such as information on previous stroke/AMI/coronary artery bypass graft surgery, assessment of left ventricular ejection fraction, coronary anatomy, etc.) are difficult to acquire and are obtained through subjective assessment, which hampers their broader application especially in the prehospital setting.

Of note, in a prospective North American cohort of patients with CS of varied aetiology, including AMI, non-AMI, and mixed aetiology of CS (as well as right, left or biventricular impairment of ejection fraction), the IABP-SHOCK II score only moderately predicted in-hospital mortality.^14^ This underlines the necessity for the development of biomarker-based prognostication models that reflect the degree of global biventricular function and multi-organ dysfunction syndrome, and are useful as ‘point-of-care’ clinical tools for the diagnosis of CS and its prognostication.^15^

There are currently over 30 prediction scores for CS which incorporate blood-based biomarkers such as lactate, creatinine, N-terminal pro-B-type natriuretic peptide (NT-proBNP), etc.^16^ Of those, the CLIP score (including cystatin C, lactate, interleukin-6 and NT-proBNP) which was also developed based on CULPRIT-SHOCK data and was externally validated in the IABP-SHOCK II cohort,^17^ provides highly accurate 30-day mortality prognostication in patients with CS following AMI. While the CLIP score incorporates common clinical biomarkers and thus offers a practical and easily clinically implementable tool, the CS4P score introduces a more molecular-level perspective of CS evolution and may thus help to identify atypical or evolving CS profiles, potentially advancing personalized clinical approaches.^17^

In the current study, the CS4P score performed better in CS patients presenting with STEMI compared with those with NSTEMI. ST-segment elevation myocardial infarction typically results from a sudden and complete occlusion of a major epicardial coronary artery, which results in a rapid decline in cardiac output, coupled with systemic hypoperfusion and elevated filling pressures, finally leading to the typical clinical picture of CS^18–20^ In contrast, while NSTEMI may involve significant myocardial injury through partial or transient coronary artery occlusion, the infarct size in these cases is generally smaller and the onset of haemodynamic deterioration is often more gradual.^21^ In these patients, CS may result not solely from extensive myocardial loss, but rather from multiple additional factors, including pre-existing left ventricular dysfunction, multivessel coronary artery disease, recurrent ischaemia, or diastolic dysfunction, processes which may be insufficiently represented by the biomarkers included in CS4P score.^22^

Cardiac arrest and CS occur frequently together, although their pathophysiological aetiologies (electrical instability vs pump failure) and clinical consequences (anoxic encephalopathy vs multisystem organ failure) often differ.^23,24^ Multivariate analysis in the CULPRIT-SHOCK study cohort identified cardiac arrest as an independent predictor of 1-year mortality.^25^ Kanhouche *et al*.^26^ found that, in STEMI patients cardiac arrest, irrespective of the presence of CS, was associated with approximately three-fold higher mortality, with this effect persisting for up to 10 months after STEMI.

In the current study, the CS4P score demonstrated statistically higher accuracy in mortality prognostication in STEMI patients without cardiac arrest and CPR as compared with those with CPR. While the prognosis of CS patients without cardiac arrest is determined primarily by shock severity, the prognosis of patients with CS who have suffered cardiac arrest is partly determined by the extent of anoxic brain injury, as predicted by arrest rhythm and location, as well as duration of resuscitation.^27^ There is no biomarker included in the CS4P score that directly reflects the degree of anoxic brain injury, which may explain the inferior prognostic performance of CS4P in STEMI patients after CPR.

The CS4P biomarker panel was established in an explorative analysis where only patients with CS due to AMI with STEMI were included. The validation of CS4P was done in the CardShock cohort incorporating CS patients irrespective of underlying aetiology, where 51.5% of CS was caused by STEMI. Cardiogenic shock can be regarded to represent the end-clinical stage of a plethora of highly different pathophysiological processes where next to coronary artery disease also myocardial inflammation, valvular heart disease or end-stage chronic heart failure may be causal. Thus, it seems to be highly challenging that one universal biomarker panel will equally reliable perform in terms of prognostication across all clinical sub-phenotypes of CS. Although the CS4P score showed overall good prognostic performance in our study cohort of CS due to AMI, its further validation in other CS forms and comparison with other biomarker-based models is warranted.

Beta-2-microglobulin is an important component of the major histocompatibility complex Class I, which is expressed on the surface of almost all nucleated cells, and is essential for immune-related communication and structural stability.^28^ In this way beta-2-microglobulin plays a key role in antigen presentation and processing, inflammation, the complement cascade, stress response, all pathophysiological processes inherent and immanent to CS development^29–32^ In our study beta-2-microglobulin exhibited the highest relative contribution to CS4P prognostication. Previous studies found that beta-2-microglobulins are elevated in coronary artery disease and are associated with a higher risk for major adverse cardiovascular events in these patients.^33^ The fact that all patients in our cohort suffered from AMI and therefore had underlying coronary artery disease may explain its higher relative contribution to CS4P based mortality prognostication.

Although both beta-2-microglobulin and liver-type FABP1 are known markers of tubular dysfunction in acute kidney injury, the CS4P score provided poor prognostication of necessity for renal replacement therapy.^34^ On contrary, the CS4P score had a high negative predictive value for renal replacement therapy, suggesting that low levels of CS4P biomarkers are associated with a lower degree and frequency of renal impairment in CS.

There are several limitations of the current study. First, we did not use the same analytical kits for CS4P biomarker determination which were used in development study. Secondly, there were no data on the degree of neurological impairment in CS patients after CPR, which could be responsible for observed results. Thirdly, the information regarding duration and course of CPR was not included in the present analysis.

### Conclusion

The CS4P score provides acceptable short-term mortality risk stratification among patients with CS due to AMI. The CS4P prediction model exhibits superior prognostication among CS patients with STEMI as compared with NSTEMI and in STEMI patients without CPR prior to hospital presentation.

## Supplementary Material

xvag010_Supplementary_Data

## Data Availability

The data underlying this article will be shared on reasonable request to the corresponding author.

## Pre-registered Clinical Trial Number

None supplied.

## References

[1] Lüsebrink E, Binzenhöfer L, Adamo M, Lorusso R, Mebazaa A, Morrow DA, et al Cardiogenic shock. Lancet 2024;404:2006–20. 10.1016/S0140-6736(24)01818-X 39550175

[2] Thiele H, Zeymer U, Akin I, Behnes M, Rassaf T, Mahabadi AA, et al Extracorporeal life support in infarct-related cardiogenic shock. N Engl J Med 2023;389:1286–97. 10.1056/NEJMoa2307227 37634145

[3] Tehrani BN, Truesdell AG, Sherwood MW, Desai S, Tran HA, Epps KC, et al Standardized team-based care for cardiogenic shock. J Am Coll Cardiol 2019;73:1659–69. 10.1016/j.jacc.2018.12.084 30947919

[4] Rivas-Lasarte M, Sans-Roselló J, Collado-Lledó E, González-Fernández V, Noriega FJ, Hernández-Pérez FJ, et al External validation and comparison of the CardShock and IABP-SHOCK II risk scores in real-world cardiogenic shock patients. Eur Heart J Acute Cardiovasc Care 2021;10:16–24. 10.1177/2048872619895230 32004078

[5] Rueda F, Borràs E, García-García C, Iborra-Egea O, Revuelta-López E, Harjola VP, et al Protein-based cardiogenic shock patient classifier. Eur Heart J 2019;40:2684–94. 10.1093/eurheartj/ehz294 31204432

[6] Thiele H, Akin I, Sandri M, Fuernau G, de Waha S, Meyer-Saraei R, et al PCI strategies in patients with acute myocardial infarction and cardiogenic shock. N Engl J Med 2017;377:2419–32. 10.1056/NEJMoa1710261 29083953

[7] Ibanez B, James S, Agewall S, Antunes MJ, Bucciarelli-Ducci C, Bueno H, et al 2017 ESC Guidelines for the management of acute myocardial infarction in patients presenting with ST-segment elevation: The Task Force for the management of acute myocardial infarction in patients presenting with ST-segment elevation of the European Society of Cardiology (ESC). Eur Heart J 2018;39:119–77. 10.1093/eurheartj/ehx393 28886621

[8] Soar J, Nolan JP, Böttiger BW, Perkins GD, Lott C, Carli P, et al European Resuscitation Council Guidelines for Resuscitation 2015: adult advanced life support. Resuscitation 2015;95:100–47. 10.1016/j.resuscitation.2015.07.016 26477701

[9] Huber K . Scores for outcome prediction in patients admitted with cardiogenic shock. Eur Heart J 2021;42:2353–5. 10.1093/eurheartj/ehab211 34110419

[10] Sleeper LA, Reynolds HR, White HD, Webb JG, Dzavík V, Hochman JS. A severity scoring system for risk assessment of patients with cardiogenic shock: a report from the SHOCK Trial and Registry. Am Heart J 2010;160:443–50. 10.1016/j.ahj.2010.06.024 20826251 PMC4229030

[11] Klein LW, Shaw RE, Krone RJ, Brindis RG, Anderson HV, Block PC, et al Mortality after emergent percutaneous coronary intervention in cardiogenic shock secondary to acute myocardial infarction and usefulness of a mortality prediction model. Am J Cardiol 2005;96:35–41. 10.1016/j.amjcard.2005.02.040 15979429

[12] Pöss J, Köster J, Fuernau G, Eitel I, de Waha S, Ouarrak T, et al Risk stratification for patients in cardiogenic shock after acute myocardial infarction. J Am Coll Cardiol 2017;69:1913–20. 10.1016/j.jacc.2017.02.027 28408020

[13] Harjola VP, Lassus J, Sionis A, Kober L, Tarvasmaki T, Spinar J, et al Clinical picture and risk prediction of short-term mortality in cardiogenic shock. Eur J Heart Fail 2015;17:501–9. 10.1002/ejhf.260 25820680

[14] Alviar CL, Li BK, Keller NM, Bohula-May E, Barnett C, Berg DD, et al Prognostic performance of the IABP-SHOCK II Risk Score among cardiogenic shock subtypes in the critical care cardiology trials network registry. Am Heart J 2024;270:1–12. 10.1016/j.ahj.2023.12.018 38190931 PMC11032171

[15] Kalra S, Ranard LS, Memon S, Rao P, Garan AR, Masoumi A, Burkhoff, et al Risk prediction in cardiogenic shock: current state of knowledge, challenges and opportunities. J Card Fail 2021;27:1099–110. 10.1016/j.cardfail.2021.08.003 34625129

[16] Wang J, Shen B, Feng X, Zhang Z, Liu J, Wang Y. A review of prognosis model associated with cardiogenic shock after acute myocardial infarction. Front Cardiovasc Med 2021;8:754303. 10.3389/fcvm.2021.754303 34957245 PMC8702644

[17] Ceglarek U, Schellong P, Rosolowski M, Scholz M, Willenberg A, Kratzsch J, et al The novel cystatin C, lactate, interleukin-6, and N-terminal pro-B-type natriuretic peptide (CLIP)-based mortality risk score in cardiogenic shock after acute myocardial infarction. Eur Heart J 2021;42:2344–52. 10.1093/eurheartj/ehab110 33647946

[18] Bentzon JF, Otsuka F, Virmani R, Falk E. Mechanisms of plaque formation and rupture. Circ Res 2014;114:1852–66. 10.1161/CIRCRESAHA.114.302721 24902970

[19] Brener SJ . Insights into the pathophysiology of ST-elevation myocardial infarction. Am Heart J 2006;151:S4–10. 10.1016/j.ahj.2006.04.007 16777509

[20] Anderson ML, Peterson ED, Peng SA, Wang TY, Ohman EM, Bhatt DL, et al Differences in the profile, treatment, and prognosis of patients with cardiogenic shock by myocardial infarction classification: a report from NCDR. Circ Cardiovasc Qual Outcomes 2013;6:708–15. 10.1161/CIRCOUTCOMES.113.000262 24221834

[21] Martínez MJ, Rueda F, Labata C, Oliveras T, Montero S, Ferrer M, et al Non-STEMI vs. STEMI cardiogenic shock: clinical profile and long-term outcomes. J Clin Med 2022;11:3558. 10.3390/jcm11123558 35743628 PMC9224589

[22] Khalid L, Dhakam SH. A review of cardiogenic shock in acute myocardial infarction. Curr Cardiol Rev 2008;4:34–40. 10.2174/157340308783565456 19924275 PMC2774583

[23] Jentzer JC, van Diepen S, Henry TD. Understanding how cardiac arrest complicates the analysis of clinical trials of cardiogenic shock. Circ Cardiovasc Qual Outcomes 2020;13:e006692. 10.1161/CIRCOUTCOMES.120.006692 32862695

[24] Zeymer U, Freund A, Noc M, Akin I, Huber K, Pöss J, et al Influence of resuscitated cardiac arrest on efficacy and safety of extracorporeal life support in infarct-related cardiogenic shock: a substudy of the ECLS-SHOCK trial. Circulation 2025;151:1752–4. 10.1161/CIRCULATIONAHA.124.073533 40523048

[25] Zeymer U, Alushi B, Noc M, Mamas MA, Montalescot G, Fuernau G, et al Influence of culprit lesion intervention on outcomes in infarct-related cardiogenic shock with cardiac arrest. J Am Coll Cardiol 2023;81:1165–76. 10.1016/j.jacc.2023.01.029 36948733

[26] Kanhouche G, Nicolau JC, de Mendonça Furtado RH, Carvalho LS, Dalçoquio TF, Pileggi B, et al Long-term outcomes of cardiogenic shock and cardiac arrest complicating ST-elevation myocardial infarction according to timing of occurrence. Eur Heart J Open 2024;4:oeae075. 10.1093/ehjopen/oeae075 39346895 PMC11430270

[27] Omer MA, Tyler JM, Henry TD, Garberich R, Sharkey SW, Schmidt CW, et al Clinical characteristics and outcomes of STEMI patients with cardiogenic shock and cardiac arrest. JACC Cardiovasc Interv 2020;13:1211–9. 10.1016/j.jcin.2020.04.004 32438992

[28] Li L, Dong M, Wang XG. The implication and significance of beta 2 microglobulin: a conservative multifunctional regulator. Chin Med J 2016;129:448–55. 10.4103/0366-6999.176084 26879019 PMC4800846

[29] Horton R, Wilming L, Rand V, Lovering RC, Bruford EA, Khodiyar VK, et al Gene map of the extended human MHC. Nat Rev Genet 2004;5:889–99. 10.1038/nrg1489 15573121

[30] Trowsdale J, Knight JC. Major histocompatibility complex genomics and human disease. Annu Rev Genomics Hum Genet 2013;14:301–23. 10.1146/annurev-genom-091212-153455 23875801 PMC4426292

[31] Barton KT, Kakajiwala A, Dietzen DJ, Goss CW, Gu H, Dharnidharka VR. Using the newer kidney disease: improving global outcomes criteria, beta-2-microglobulin levels associate with severity of acute kidney injury. Clin Kidney J 2018;11:797–802. 10.1093/ckj/sfy056 30524714 PMC6275448

[32] Shirakabe A, Matsushita M, Shibata Y, Shighihara S, Nishigoori S, Sawatani T, et al Organ dysfunction, injury, and failure in cardiogenic shock. J Intensive Care 2023;11:26. 10.1186/s40560-023-00676-1 37386552 PMC10308671

[33] Möckel M, Muller R, Searle J, Slagman A, De Bruyne B, Serruys P, et al Usefulness of beta2-microglobulin as a predictor of all-cause and nonculprit lesion-related cardiovascular events in acute coronary syndromes (from the PROSPECT study). Am J Cardiol 2015;116:1034–40. 10.1016/j.amjcard.2015.07.017 26254706

[34] Zhao M, Liu J, Zhuang H, Qiu Y, He Z, Lin J, et al Beta 2-microglobulin is an independent risk marker of acute kidney injury in adult patients with hemophagocytic lymphohistiocytosis. J Nephrol 2024;37:1317–25. 10.1007/s40620-024-01949-0 38735000 PMC11405466

