# Supplementary material for: Prognostic performance of cardiogenic shock 4 proteins prediction model in infarct-related cardiogenic shock
Source: ESC Heart Fail. 2026 Jan 20;13(2):xvag010. doi: 10.1093/eschf/xvag010 (PMC13001806; doi:10.1093/eschf/xvag010)
Supplement: xvag010_Supplementary_Data [file xvag010_supplementary_data.docx]

**Supplementary material**

**Supplementary Figure 1.** CS patients presenting as NSTEMI without cardiopulmonary resuscitation prior to study inclusion


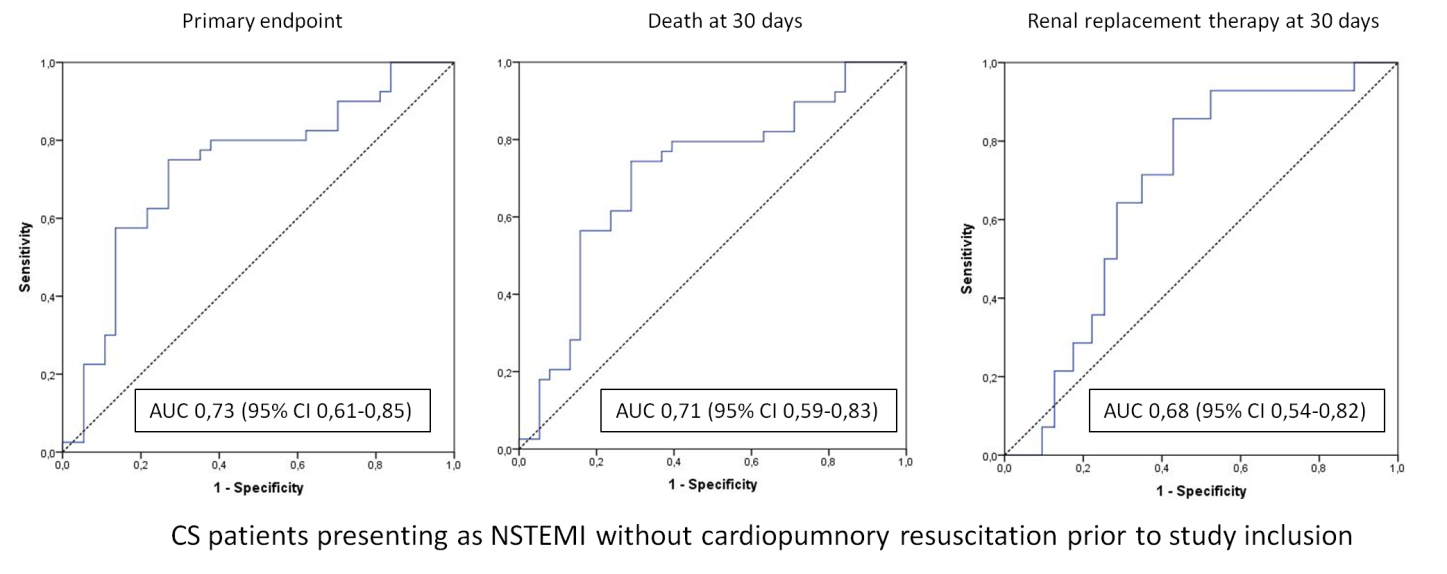


**Supplementary Figure 2.** CS patients presenting as NSTEMI with cardiopulmonary resuscitation prior to study inclusion


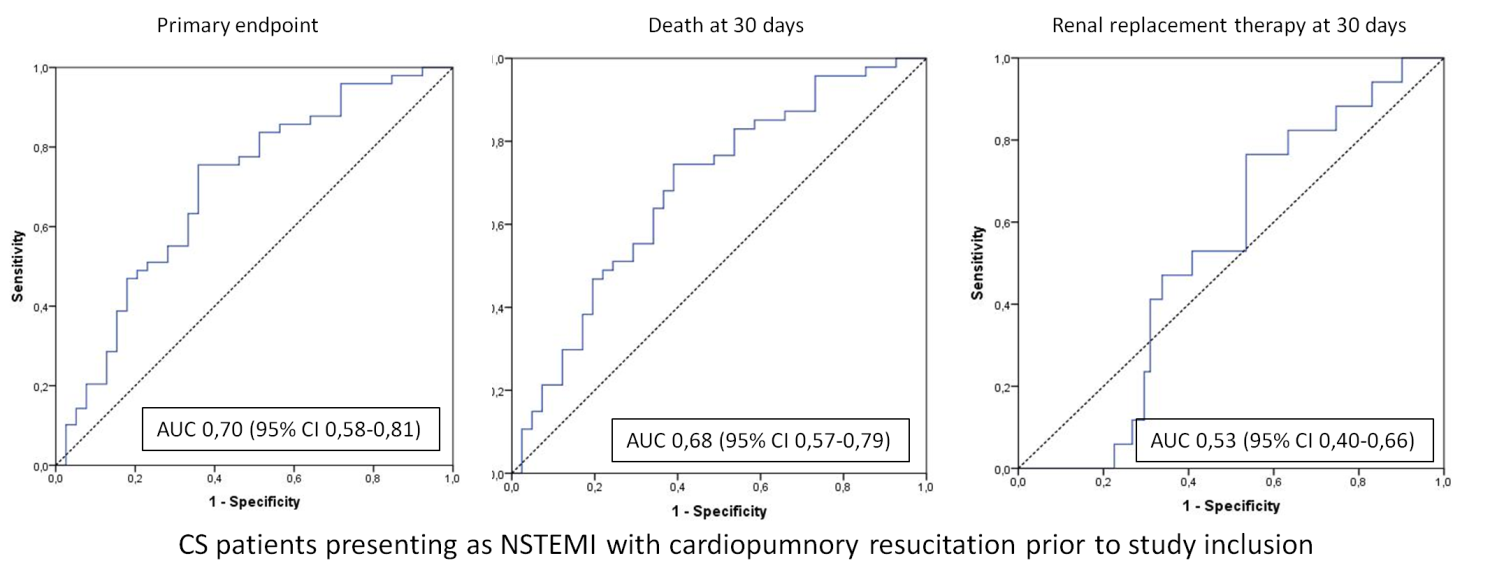


**Supplementary Figure 3.** Kaplan–Meier estimated cumulative event rate for the 30 day mortality depicted for patients stratified by tertiles of the CS4P predicted probability adjusted for age, randomisation group and necessity for mechanical circulatory support.


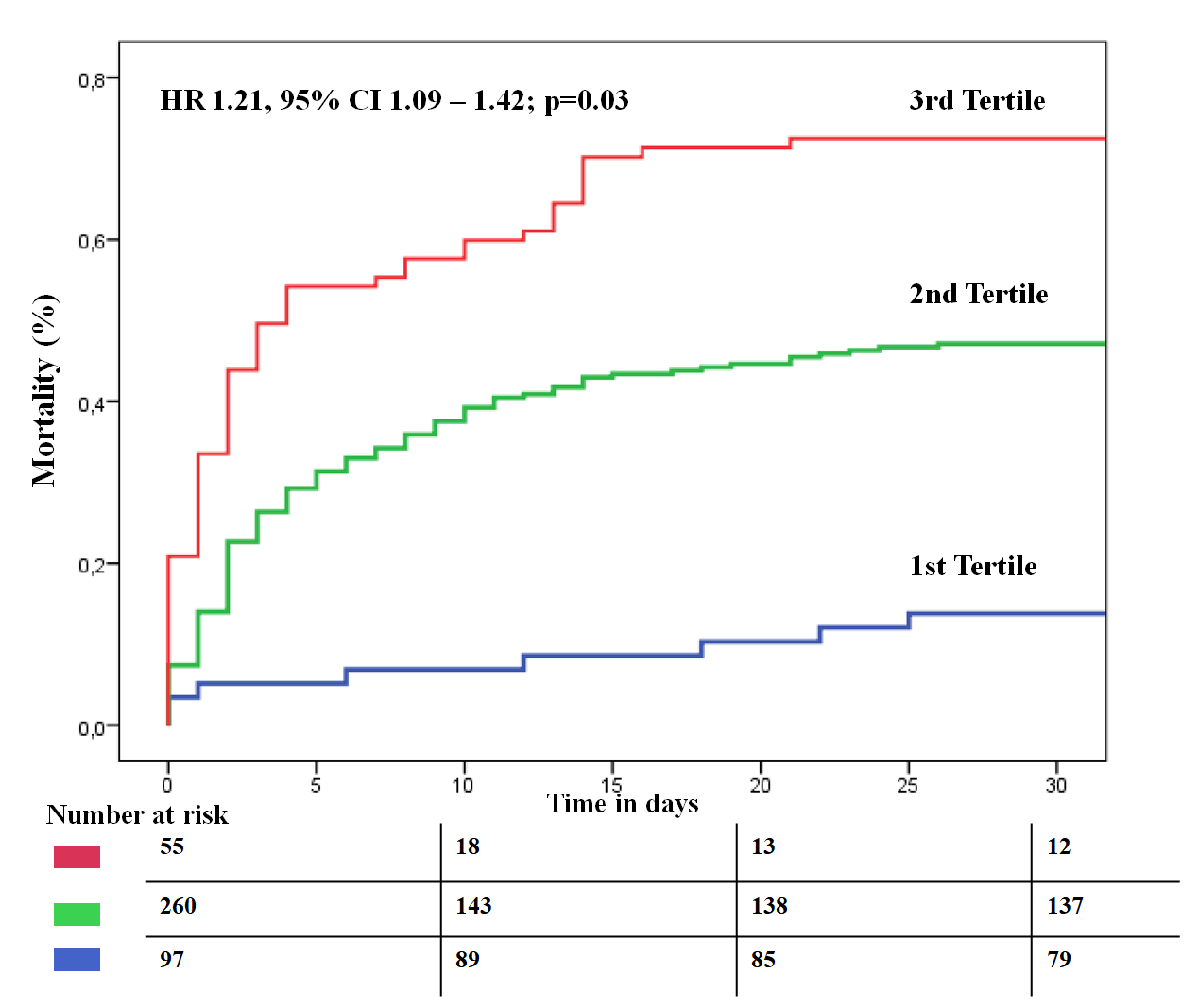


**Supplementary Figure 4.** ROC analysis of CS4P prognostication of death and necessity for renal replacement therapy at 30 days follow-up in the entire study cohort

**
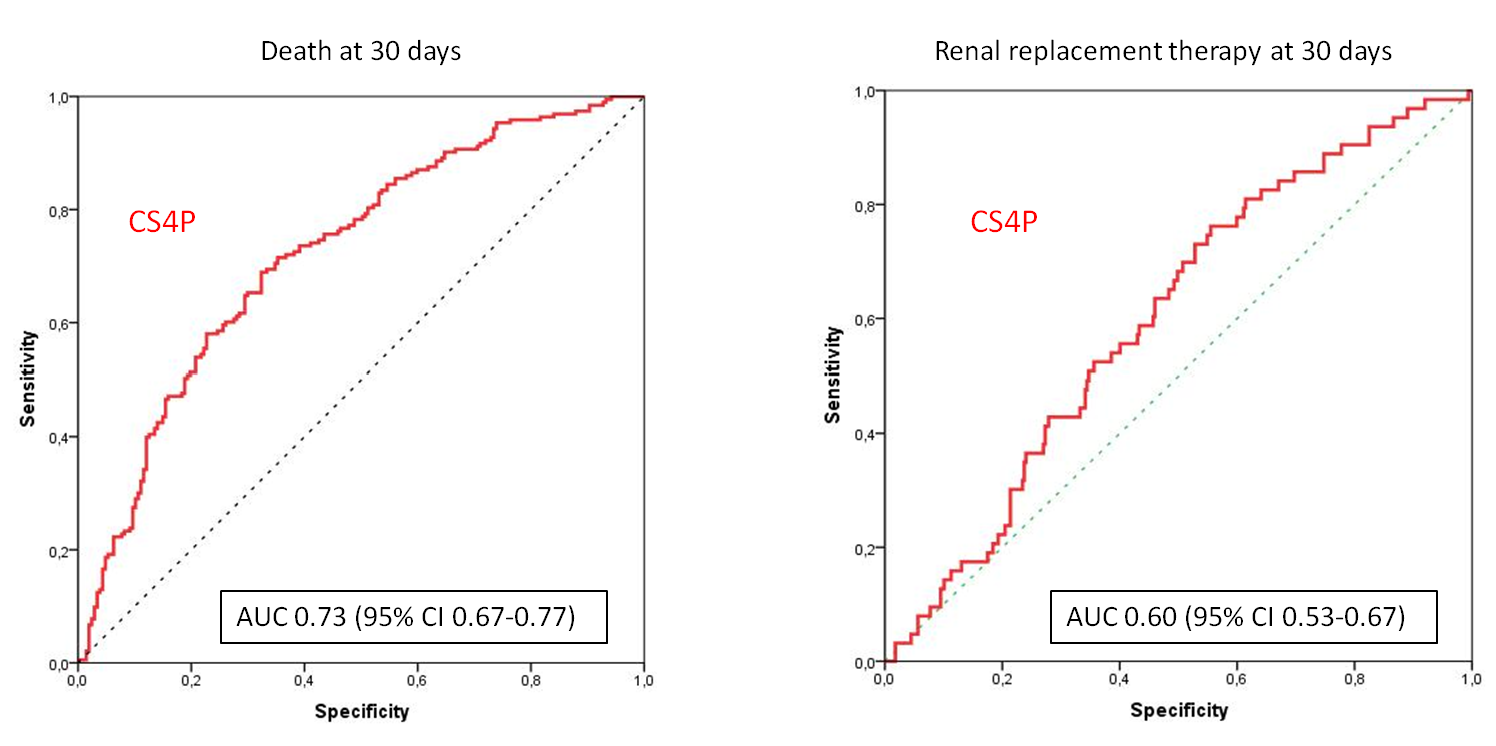
**

**Supplementary Table 1.** Differences in clinical characteristics between patients from CULPRIT-SHOCK trial who were included in our study versus non-included patients

| **Baseline characteristics of CS patients from CULPRIT-SHOCK trial (n=686)** | **Included patients (n=412)** | **Non-included patients (n=274)** | ***p*** |
| --- | --- | --- | --- |
| Age, years (mean ± SD) | 68 ± 10 | 69 ± 11 | 0.72 |
| Male-no./total no. (%) | 310/412 (75.2 %) | 200/274 (72.9 %) | 0.62 |
| BMI, kg/m² ( mean ± SD) | 27.5 ± 4.6 | 26.9 ± 4.5 | 0.09 |
| Hypertension - no./total no. (%) | 248/412 (60.2 %) | 161/274 (58.7 %) | 0.67 |
| Diabetes mellitus - no./total no. (%) | 135/412 (32.7 %) | 86/274 (31.3 %) | 0.60 |
| Previous myocardial infarction - no./total no. (%) | 61/412 (14.8 %) | 55/274 (20.1 %) | 0.10 |
| Dyslipidemia - no./total no. (%) | 126/412 (30.5 %) | 106/274 (38.6 %) | 0.06 |
| Previous PCI - no./total no. (%) | 77/412 (18.7 %) | 52/274 (18.9 %) | 0.92 |
| Known peripheral artery disease - no./total no. (%) | 44/412 (10.6 %) | 39/274 (14.2 %) | 0.22 |
| Previous CABG - no./total no. (%) | 21/412 (5.1 %) | 12/274 (4.3 %) | 0.81 |
| Atrial fibrillation - no./total no. (%) | 43/412 (10.4%) | 36/274 (13.1 %) | 0.51 |
| Previous stroke - no./total no. (%) | 25/412 (6.1 %) | 26/274 (9.4 %) | 0.12 |
| Known chronic kidney failure (GFR<30 ml/min) - no./total no. (%) | 29/412 (7.0 %) | 17/274 (6.2 %) | 0.67 |
| CPR 24 h before randomization - no./total no. (%) | 216/412 (52.4 %) | 152/274 (55.6 %) | 0.46 |
| Serum lactate pre-PCI, mmol/L ( mean ± SD) | 4.6 ± 2.5 | 4.8 ± 2.7 | 0.35 |
| Creatinine, µmol/L (total no.) | 123 ± 67 (393) | 127 ± 55 (266) | 0.65 |
| Acute left ventricular ejection fraction, % ( mean ± SD) | 34 ± 13 | 31 ± 9 | 0.17 |
| Mechanical circulatory support (any) - no./total no. (%) | 113/412 (27.4 %) | 79/274 (28.8 %) | 0.76 |
| Mechanical ventilation - no./total no. (%) | 335/412 (81.3 %) | 219/274 (80.0 %) | 0.79 |
| Catecholamine therapy - no./total no. (%) | 367/412 (89.0 %) | 242/274 (88.3 %) | 0.53 |
| Culprit-lesion only - no./total no. (%) | 205/412 (49.7 %) | 136/274 (49.6 %) | 0.15 |
